# Glycosaminoglycans Are Interactants of Langerin: Comparison with gp120 Highlights an Unexpected Calcium-Independent Binding Mode

**DOI:** 10.1371/journal.pone.0050722

**Published:** 2012-11-30

**Authors:** Eric Chabrol, Alessandra Nurisso, Antoine Daina, Emilie Vassal-Stermann, Michel Thepaut, Eric Girard, Romain R. Vivès, Franck Fieschi

**Affiliations:** 1 Groupe Membrane & Pathogens, Institut de Biologie Structurale, Université Joseph Fourier, Grenoble, France; 2 UMR 5075, CNRS, Grenoble, France; 3 Departement des sciences du vivant, CEA, Grenoble, France; 4 Département de Pharmacochimie, Université de Genève, Genève, Switzerland; 5 Molecular Modeling Group, Swiss Institute of Bioinformatics, Lausanne, Switzerland; 6 Groupe SAGAG, Institut de Biologie Structurale, Université Joseph Fourier, Grenoble, France; 7 Groupe ELMA, Institut de Biologie Structurale, Université Joseph Fourier, Grenoble, France; 8 Institut Universitaire de France, Paris, France; University of Patras, Greece

## Abstract

Langerin is a C-type lectin specifically expressed in Langerhans cells. As recently shown for HIV, Langerin is thought to capture pathogens and mediate their internalisation into Birbeck Granules for elimination. However, the precise functions of Langerin remain elusive, mostly because of the lack of information on its binding properties and physiological ligands. Based on recent reports that Langerin binds to sulfated sugars, we conducted here a comparative analysis of Langerin interaction with mannose-rich HIV glycoprotein gp120 and glycosaminoglycan (GAGs), a family of sulfated polysaccharides expressed at the surface of most mammalian cells. Our results first revealed that Langerin bound to these different glycans through very distinct mechanisms and led to the identification of a novel, GAG-specific binding mode within Langerin. In contrast to the canonical lectin domain, this new binding site showed no Ca^2+^-dependency, and could only be detected in entire, trimeric extracellular domains of Langerin. Interestingly binding to GAGs, did not simply rely on a net charge effect, but rather on more discrete saccharide features, such as 6-O-sulfation, or iduronic acid content. Using molecular modelling simulations, we proposed a model of Langerin/heparin complex, which located the GAG binding site at the interface of two of the three Carbohydrate-recognition domains of the protein, at the edge of the a-helix coiled-coil. To our knowledge, the binding properties that we have highlighted here for Langerin, have never been reported for C-type lectins before. These findings provide new insights towards the understanding of Langerin biological functions.

## Introduction

Langerin is a C-type lectin receptor highly expressed in Langerhans cells (LCs), a subset of dendritic cells, which reside in skin epidermis and mucosal epithelium. From the N- to the C-terminus, Langerin is composed of a short cytoplasmic region, a unique transmembrane domain and a large extracellular domain (ECD) subdivided into a neck domain and a C-terminal carbohydrate-recognition domain (CRD). Initially identified as a molecular marker of LCs (recognized by the LC-specific DCGM4 monoclonal Antibody) [1], Langerin initially caught attention, a decade ago, for its unique ability to promote, by itself, the formation of a specific organelle, only present in LCs, the Birbeck Granule [2]. More recently, this feature was further highlighted by the observation that Langerin was able to prevent HIV transmission to T-cells following direct interaction with gp120 and internalization of the virus within Birbeck Granule for elimination [3]. The implication of Langerin in the prevention of HIV transmission strongly contrasts with the fate of HIV particles interacting with DC-SIGN, another C-type lectin receptor of the same family [4]. Indeed, DC-SIGN, which is present at the surface of another subtype of dendritic cells, is largely described as an important factor promoting trans-infection of HIV particles from DCs to T-cells [5], [6] and is therefore considered as critical in the initial steps of HIV transmission. Langerin-expressing Langerhans cells are present in epidermis, the upper layer of skin and mucosa and are therefore the first cell subsets encountering the virus while DC-SIGN, expressed in immature interstitial DCs, is present in dermis and in the deeper layer of mucosa [7]. DC-SIGN has become a target for potential microbicides for many chemical consortiums which intend to develop inhibitors of the initial step of HIV transmission [8]–[14]. However, it seems that, besides being a powerful DC-SIGN inhibitor, the perfect compound should also have no effect on Langerin function to preserve the efficacy of the natural mucosal barrier towards HIV genital infection. A research consortium to which we belong has been jointly working along these lines with some preliminary successes [13], [15].

To support these developments, but also to better understand the biological role of Langerin, a good knowledge of its binding properties together with the identification of natural and potentially physiological ligands is essential. Some glycan arrays studies have already shed light on the specific properties of Langerin glycan recognition [16], [17], [18].

Within its Ca^2+^ binding site, Langerin is able to recognize oligosaccharides with terminal Man or GlcNAc and also, with some restriction, oligosaccharides involving terminal fucose linked to galactose [16], [18]. DC-SIGN can bind to internal Man within large oligosaccharides and can also recognize a wide variety of fucose from Lewis antigen derivatives [19]. Indeed, Langerin and DC-SIGN share the ability to bind high mannose structures, as found on the HIV gp120 envelope protein.

T. Feizi, the Nobel laureate R. Steinman *et al.* have been the first to highlight the specificity of the murine Langerin towards sulfated sugars [20]. This study describes the specificity towards Lewis x analogs, which harbor terminal galactoses sulfated at position 6, whereas no recognition was observed with sugars sulfated at position 3 (in contrast with selectin). Moreover, sulfated dextrans also bind Langerin, suggesting that sulfated glucose polymers could be ligands as well. Tateno *et al.* recently confirmed such a specific recognition of 6-sulfated galactose by Langerin [21] and explored the binding properties of Langerin for keratan sulfate, which is a naturally Gal-6-sulfated polysaccharide of the glycosaminoglycan (GAG) family. Indeed, apart from HIV, this study proposed keratan sulfate as a potential physiological binding partner for Langerin.

From these preliminary observations, we postulated that other sulfated GAGs (such as heparan sulfate and chondroitin sulfate) might be potential ligands for Langerin. In this work, we analysed the binding properties of Langerin towards gp120 HIV envelope protein as well as the recognition of a large set of GAGs. From these comparative analyses, we firstly demonstrated that Langerin is able to bind to a broad range of GAGs with a marked preference for heparin and heparan sulfate. More surprisingly, we observed that gp120 and GAG recognition are based on totally different modes of interactions. Through its high mannose glycans, gp120 directly interacts with the Ca^2+^ dependent-binding site of the carbohydrate domain, whereas binding to GAGs appears to be totally Ca^2+^ independent. In addition, while Langerin isolated monomeric CRD is able to recognize gp120, the trimeric form of the entire extracellular domain, including CRD and neck domain, is required for the interaction with GAGs. Molecular modeling simulation was used to identify putative GAG binding sites within the protein and highlighted a most probable binding site, which location was in agreement with Ca^2+^ independency and oligomeric requirements for intermolecular recognition.

To our knowledge, such structural requirements for sugar binding have never been reported for a C-type lectin receptor (CLR). As such, our study describes a novel binding mode, and addresses new questions regarding the physiology of LCs in epidermis and mucosa, which contain large amount of GAGs.

## Results

### Analysis of Langerin/heparin Interaction by SPR

**Figure 1:**
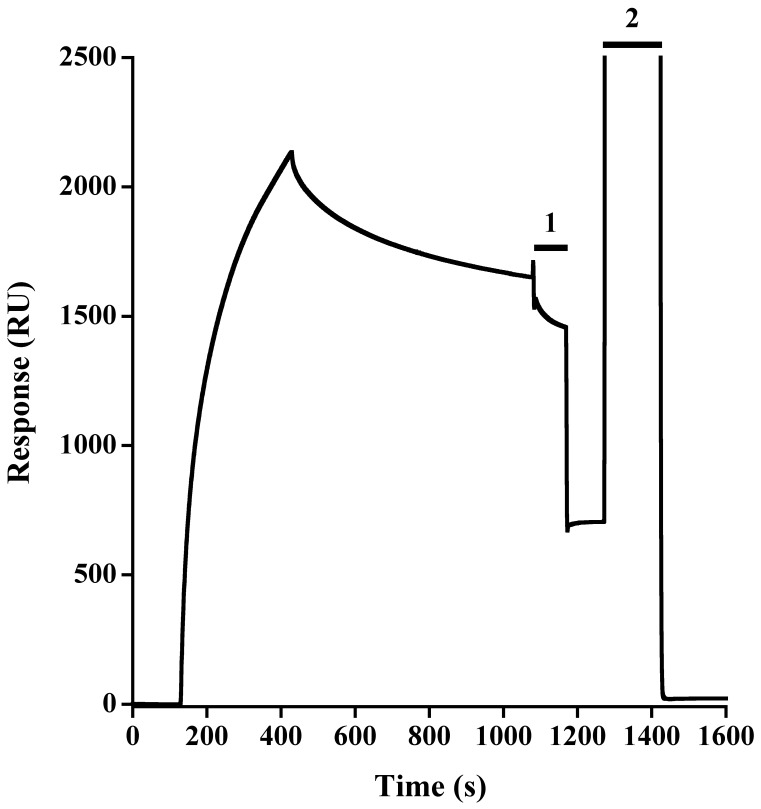
Langerin ECD interaction onto heparin. Surface was functionalized with heparin 6 kDa. 100 µL Langerin ECD at 500 nM are injected onto the surface in a Ca^2+^ containing running buffer. Two modes of surface regenerations are tested, 1: Injection of 30 µL of 50 mM EDTA. 2: Injection of 50 µL of 350 mM MgCl_2_.

Langerin is a lectin able to recognize various sulfated carbohydrates. Among natural carbohydrate polymers harboring sulfate groups, GAGs are widely distributed in epithelia where Langerhans cells are present [22] and could thus candidate for Langerin natural ligands. To investigate this, we first analysed binding of Langerin to heparin by SPR. On a CM4 sensor chip functionalised with saturating amounts of streptavidin, we immobilized 6 kDa (Figure 1) and 15 kDa heparin (not shown) onto different flow cells up to 30 RU. Langerin ECD interacted with both heparin surfaces but surface regeneration with EDTA was not complete, suggesting that the interaction between Langerin and heparin is more complex than classical C-type lectin carbohydrate interactions, which are often strictly Ca^2+^ dependent. However, complete regeneration was finally obtained with a flash injection of MgCl_2._ To characterize further the binding properties of Langerin to heparin, we conducted a parallel comparison with the Langerin/gp120 interaction, which was expected to be Ca^2+^ dependent as for classical C-type lectin-glycoconjugate complex.

### Comparison of Langerin Interaction Properties Towards HIV-1 gp120 and Heparin

**Figure 2:**
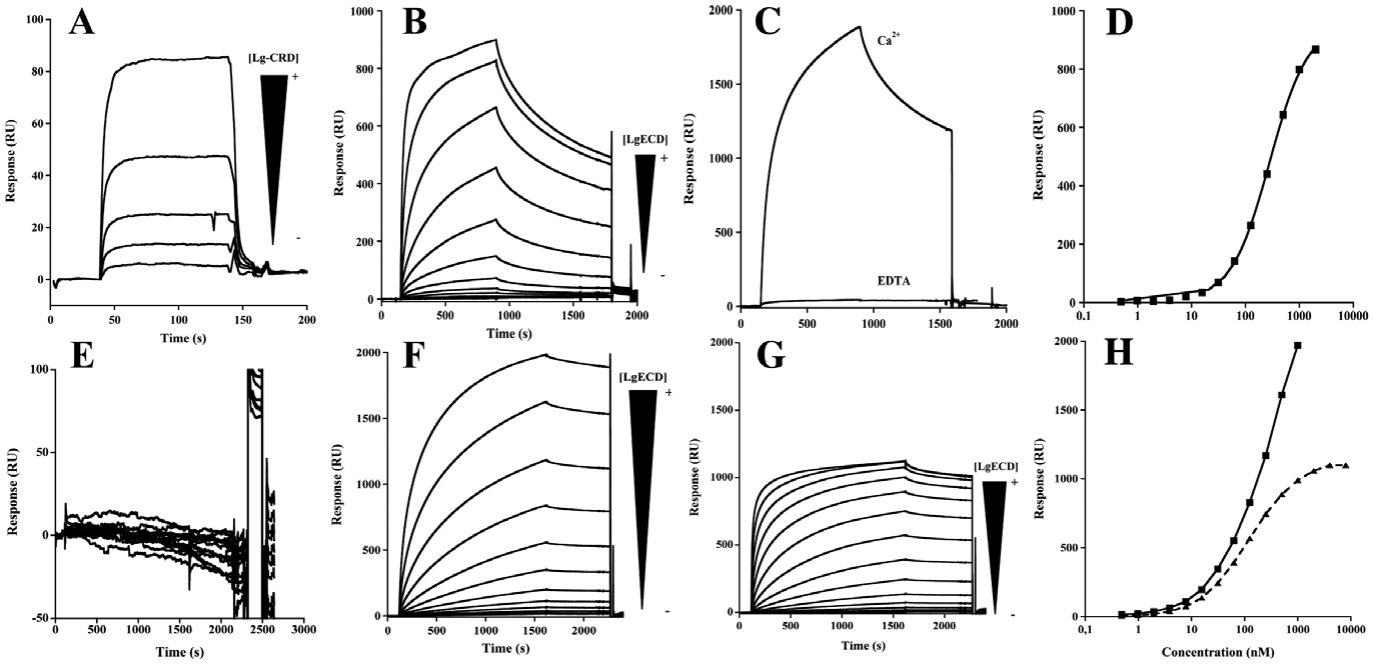
Comparison of heparin and gp120 binding mode to Langerin. A) Overlay of sensorgram showing Langerin CRD interaction onto gp120_YU2_ functionalized surface in Ca^2+^ buffer. Langerin CRD concentration range is from 400 µM to 12,5 µM with 2 times serial dilution. B) Overlay of sensorgram showing Langerin ECD interaction onto gp120_YU2_ functionalised surface in calcium buffer. The concentration range of Langerin ECD is from 2 µM to 7,8 nM with 2 times serial dilution. C) SPR sensorgram of Langerin ECD interaction onto gp120_YU2_ functionalised surface in calcium and EDTA buffer. Langerin ECD injection has been performed at 500 nM concentration of protein. D) SPR binding analysis of gp120 interaction as a function of Langerin ECD concentration. E) Overlay of sensorgram showing Langerin CRD interaction onto biotinylated 6 kDa heparin functionalised surface in calcium buffer. Langerin CRD concentration was from 100 µM to 1.6 µM with 2-fold serial dilution. F) Overlay of sensorgram showing of Langerin ECD interaction onto biotinylated 6 kDa heparin functionalised surface in calcium buffer. The concentration range of Langerin ECD is from 1 µM to 0,49 nM with 2 times serial dilution. G) SPR titration experiment of Langerin ECD interaction onto biotinylated 6 kDa heparin functionalised surface in EDTA buffer. The concentration range of Langerin ECD is from 8 µM to 0,488 nM with 2 times serial dilution. H) Overlay of sensorgram showing Langerin ECD on 6kDa heparin surface in calcium buffer (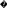) and in EDTA buffer (←).

Comparison of Langerin interaction for heparin and gp120 clearly states that both ligands interact through different binding modes. Langerin interaction with the gp120 surface displays the canonical behavior awaited for C-type lectin receptors (CLRs) towards classical glycoconjugates. Indeed, CRD is able to bind to gp120 and represents the domain unit of recognition binding (Figure 2A) and a clear affinity improvement is observed, through an avidity effect, using the whole trimeric extracellular domain (Figure 2B). Due to the complexity of the Lg ECD multivalent interaction, no *K_d_* could be determined with enough accuracy. However, from the range of concentrations tested, an EC_50_ was estimated around 282±3 nM for the interaction with the gp120 surface (Figure 2D). In contrast, sensorgrams obtained for Lg CRD binding to the gp120 surface could be fitted to a 1∶1 Langmuir binding model and yielded a *K_d_* of 25 µM. These data suggest that the avidity effects triggers an increase in affinity of roughly a hundred-time. Finally, as expected, the interaction is totally calcium dependent. This was confirmed by injecting Lg ECD with either 4 mM Ca^2+^ or 1 mM EDTA in the running buffer (Figure 2C). Subsequently, EDTA was used to regenerate the surface between each protein injection, for analysis of both Langerin CRD and ECD (Figure 2A and 2B).

On the contrary, the Langerin/heparin interaction obeys to unconventional properties that, to our knowledge, have never been reported for CLRs. Firstly, no interaction is observed using the CRD domain while strong binding to heparin is observed with Lg ECD (Figure 2E and 2F). This suggests that the CRD is not the minimal binding unit for heparin but that the interaction site is created only upon formation of the Langerin oligomer. Secondly, comparison of the interaction in presence of Ca^2+^ or EDTA in the running buffer shows that the Lg ECD/heparin complex occurs, surprisingly, in both conditions (Figure 2F and 2G). From both series of experiments, we generated a titration curve that highlights strong differences in binding properties depending on the conditions used. In presence of EDTA, we were able to perform a complete titration and to reach the saturation with an EC_50_ of 150±26 nM. In contrast, no saturation of the binding could be achieved in presence of Ca^2+^. This suggests a more complex binding mechanism that takes place in presence of calcium, where additional events participating to the Lg ECD/heparin interaction most likely occur (Figure 2H). Therefore, we decided to focus on the specific Lg ECD/heparin interaction observed in presence of EDTA. On its own, this Ca^2+^-independent binding represents a new type of interaction never reported for Langerin.

### Characterization of Langerin Interaction with Glycosaminoglycans

**Figure 3:**
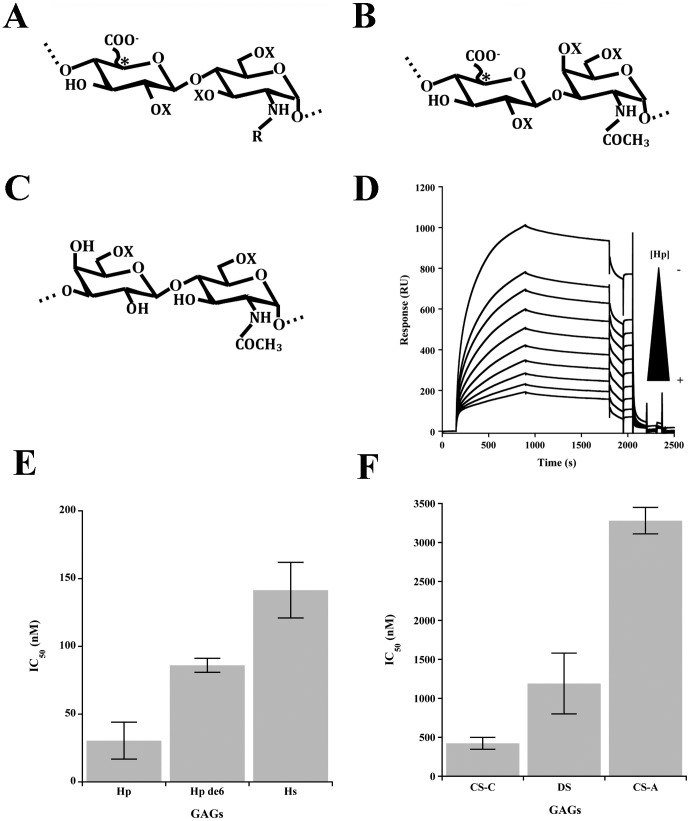
Interaction properties of Langerin to different glycosaminoglycans. (A-C) Structure of glycosaminoglycan disaccharides. Disaccharide units for heparin/HS (A), CS/DS (B) or KS (C). R = COCH_3_ or SO_3_H; X = H or SO_3_. The star indicates presence of glucuronic or iduronic acid C-5 epimers. D) SPR inhibition experiment of Langerin ECD/heparin interaction by 15 kDa heparin. Langerin ECD concentration was 100 nM and the heparin concentration range was from 7.8 nM to 2 µM with 2-fold serial dilution factor. E) IC_50_ values obtained with heparin, heparin treated with HSulf2 and HS. F) Histogram representation of CS/DS IC_50_. SPR inhibition experiment of Langerin ECD/heparin interaction by 15 kDa heparin. Langerin ECD concentration was fixed at 100 nM and the heparin concentration range was from 7.8 nM to 2 µM with 2-fold serial dilution factor. C) Histogram representation of GAGs IC_50_.

GAGs are a family of complex polysaccharides characterised by a repeating disaccharide unit comprising a N-substituted hexosamine and an uronic acid. According to the nature of the amino sugar, 2 main subfamilies can be defined. Galactosaminoglycans include galactosamine-containing Chondroitin Sulfate (CS) and Dermatan Sulfate (DS) that can be distinguished by the nature of their uronate: either exclusively glucuronic acid (GlcA) for CS or GlcA and a proportion of its C-5 epimer iduronic acid (IdoA) for DS. For glucosaminoglycans, the amino-sugar is a glucosamine that can either be associated to a GlcA (Hyaluronic Acid, HA), or a mix of GlcA/IdoA (Heparan Sulfate, HS and Heparin, Hp), or a galactose (Gal) residue (Keratan Sulfate, KS). With the exception of HA, GAG disaccharide units can be further modified by addition of O-sulfate groups: at C-6 of Gal for KS, at C-2 of IdoA, C-6 of GlcNAc/GlcNS and occasionally C-3 of GlcNS for HS/Hp and at C-4/C-6 of GalNAc and C-2 of IdoA for CS/DS. According to sulfation patterns, CS has been sub-categorized into CS-A (preferentially 4-O-sulfated) and CS-C (preferentially 6-O-sulfated) (See Figure 3A to 3C for structure description of various GAGs).

In this study, we have assessed the ability of Lg-EC to bind to various GAGs in order to identify specific saccharide features required for the interaction. Binding properties of Lg-EC towards other GAGs was assessed by competition assays. For this, Lg-EC was pre-incubated with concentration series of free GAGs, prior to injection onto the heparin functionalised surface (Figure 3D). Maximal responses (RUmax) obtained from the sensorgrams (for competition with free Hp, see Figure 3D) were then used to calculate IC_50_s for each tested GAGs (Figure 3E and 3F). Results, first indicated that Langerin preferentially binds to HS-type GAGs, as free Hp and HS were found to be the most potent inhibitors, with IC50s of 30.45±13.5 nM and 141.5±20.5 nM, respectively. CS/DS-type GAGs showed some inhibition, although to a much lower level. Interestingly, great discrepancies could be observed between these samples. CS-C was found to be the best inhibitor (IC50 of 423±76 nM), followed by DS (IC50 1.2±0.39 µM), and CS-A (IC50 of 3.3±0,17 µM). Finally, in our hands, KS failed to inhibit Lg-EC/hp interaction in the range of concentration tested (data not shown).

We then compared these binding data to the structural information obtained on these GAGs by disaccharide analysis (Table 1). Interestingly, all CS/DS samples showed very similar levels of overall sulfation, indicating that binding activity could not simply be related to a net charge effect. Although CS-A and CS-C are very closely structurally related, the latter was found to be 10 times more potent an inhibitor of Lg-EC/hp interaction than CS-A. Disaccharide analysis of these 2 samples revealed that CS-C showed a greater content in 6-O- sulfation and 2-O-sulfation, but was the least 4-Osulfated, suggesting a possible contribution of 2-O and/or 6-O sulfates in binding to Lg-EC, but not 4-O-sulfates. Surprisingly, DS inhibited Lg-EC/Hp interaction to an intermediate level, despite being the least 6-O-sulfated of all 3 samples and having a very low level of 2-O-sulfation. DS being naturally enriched in IdoA, this suggest that IdoA may be of importance for the interaction and could compensates for the lower sulfation content as it has been previously described [23].

**Table 1:** Disaccharide analysis of GAGs.

Disaccharide	Hp	Hp de6S	CS-A	CS-B	CS-C	Disaccharide
ΔHexA - GlcNAc	5.2	4.6	11	9.5	4.3	ΔHexA - GalNAc
ΔHexA - GlcNAc,6S	4.1	3.9	46.8	78.6	24.2	ΔHexA - GalNAc,4S
ΔHexA - GlcNS	6.1	10.1	41	6	52.5	ΔHexA - GalNAc,6S
ΔHexA - GlcNS,6S	12.6	9.8	0	1.2	0	ΔHexA,2S - GalNAc,4S
ΔHexA,2S - GlcNS	9.4	57.4	0.5	4.3	2.5	ΔHexA - GalNAc,4S,6S
ΔHexA,2S - GlcNS,6S	60.7	11.7	0.6	0.4	16.5	ΔHexA,2S - GalNAc,6S
ΔHexA,2S - GlcNAc	1.9	2.5				
*Sulfate/dp2*	2.4	1.9	0.9	1	1.1	*Sulfate/dp2*
*N-sulfation*	88.8	89	0.6	1.7	16.5	*2-O-sulfation*
*2-O-sulfation*	72	71.6	47.3	84.1	26.7	*4-O-sulfation*
*6-O-sulfation*	77.4	25.4	42.1	10.7	71.5	*6-O-sulfation*

For determination of GAG composition, heparin and CS samples were exhaustively depolymerised (with heparinases I, II, III and chondroitinase ABC, respectively), and the resulting disaccharides were resolved by SAX-HPLC, using a NaCl gradient calibrated with authentic standards.

Further structure/activity information could be obtained from the competition assays performed with Hp and HS. Again, HS showed inhibitory properties fairly close to that of Hp, despite being significantly less sulfated. This supported further the importance of sulfation pattern rather than net charge for the interaction. More interestingly, we found that treatment of Hp with HSulf-2 resulted in a 186% decrease of its inhibitory properties. HSulf-2 is an extracellular 6-O-sulfatase that specifically targets HS or Hp at the level of [IdoA(2S)-GlcNS(6S)] trisulfated disaccharides. Accordingly, disaccharide analysis data showed a ∼80% reduction of this disaccharide upon HSulf-2 treatment. These data highlight the importance of such disaccharide motif for Lg-EC/Hp interaction.

### Surface Mapping and EADock DSS Runs: Identification of Potential Heparin Interacting Areas on the Langerin ECD Target Protein

**Figure 4:**
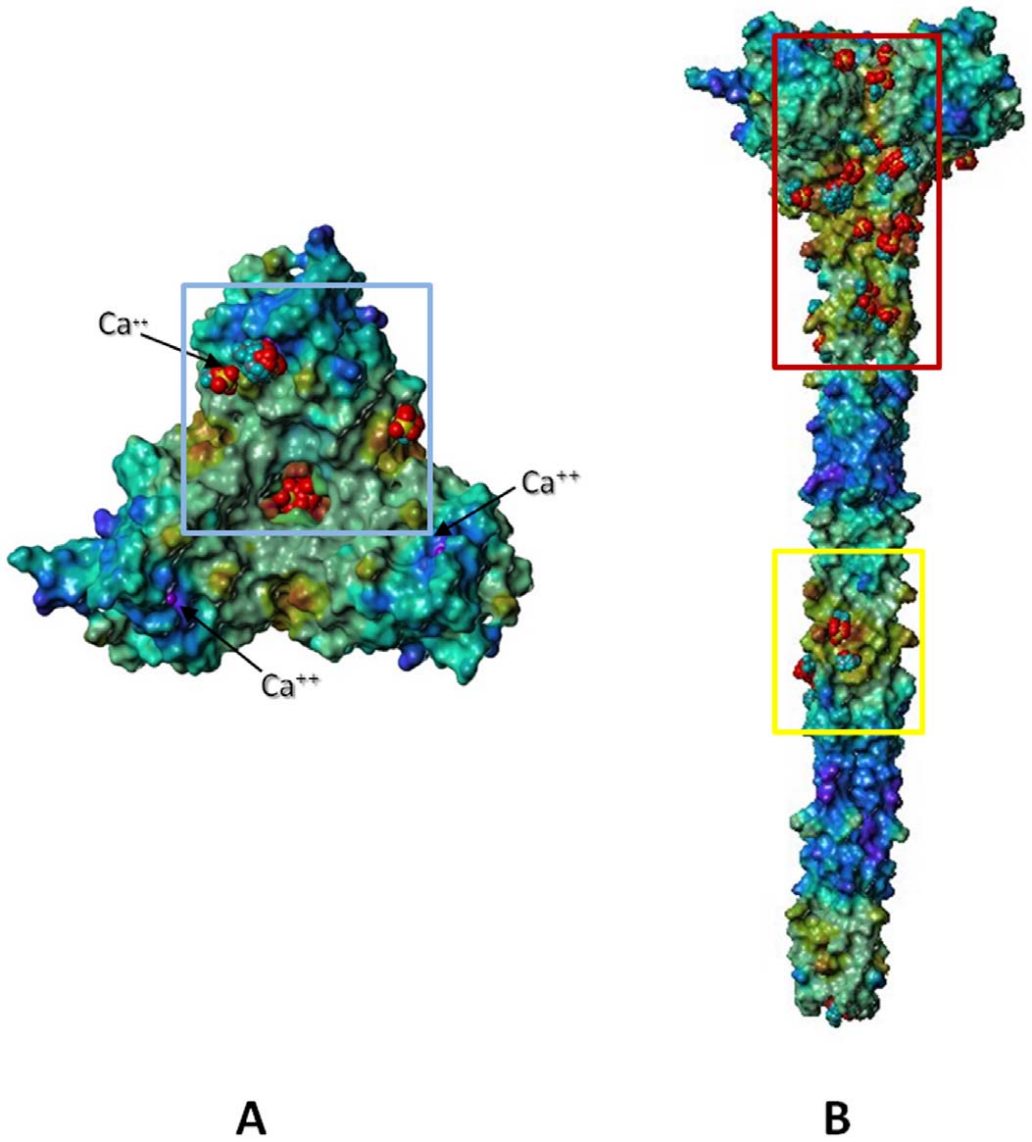
Three-dimensional model of langerin ECD and potential heparin docking sites. The human Langerin ECD is represented by its Connolly surface, color-coded according to the molecular electrostatic potential (from blue for negative to red for positive areas). The most probable regions for interactions with heparin are indicated with colored boxes populated with methylsulfate docking solutions, represented in *space fill* (5A: top view; 5B: side view).

Connolly surface of the Langerin ECD, obtained through a meticulous merging of experimental and computational structural data, was color-coded according to the molecular electrostatic potential (Figure 4A–B). Three main positively charged areas were identified. The first one refers to the cavity created by the spatial arrangement of the three Carbohydrate Recognition Domains (CRDs) characterizing Langerin, rich in Lys residues (Figure 4A, blue box). Two other positively charged zones, repeated three times over the protein surface because of the symmetric nature of Langerin, are formed by two CRDs. In particular, the second region consists in a groove formed by the α2 helix of one CRD and α1 helix of the adjacent CRD, together with the closer loops and part of the α-helix coiled-coil parts of both CRDs (Figure 4B, red box). Part of the α-helix coiled-coil region of Langerin also accommodates the third and less extended positively charged area (Figure 4B, yellow box).

To gain more insight into the possible binding of sulfated glycosaminoglycans (GAG), a methylsulfate probe was docked towards the Langerin ECD structure with the EADock DSS program, known to be suited for blind docking simulations [24]. Docking results, evaluated through a CHARMM-based energy function and clustered at 2 Å-rmsd, were visualized in the same referential as the Connolly surface. The great majority of methylsulfates were predicted to be well recognized by the positively charged areas surrounded by the red box (76.1% of all solutions) (Figure 4B) whereas the little cavity depicted by the blue box accommodates few methylsulfate fragments (5.5% of all solutions) (Figure 4A). These converging results, obtained by two unrelated computational approaches, represent robust starting points for locating the most probable areas of intermolecular recognition between Langerin and heparin fragments.

### Langerin and Heparin Recognition: Flexible Docking and Modeling of the Decameric Heparin Chain

One main and extended region was considered for Autodock3 calculations. This one includes both the blue and red boxes as shown in the Figure 4. Its dimension allowed accounting for only one of the three identical sites created by the symmetrical nature of Langerin. Heparin fragments were chosen instead of a long heparin chain to allow full ligand flexibility during calculations.

**Figure 5:**
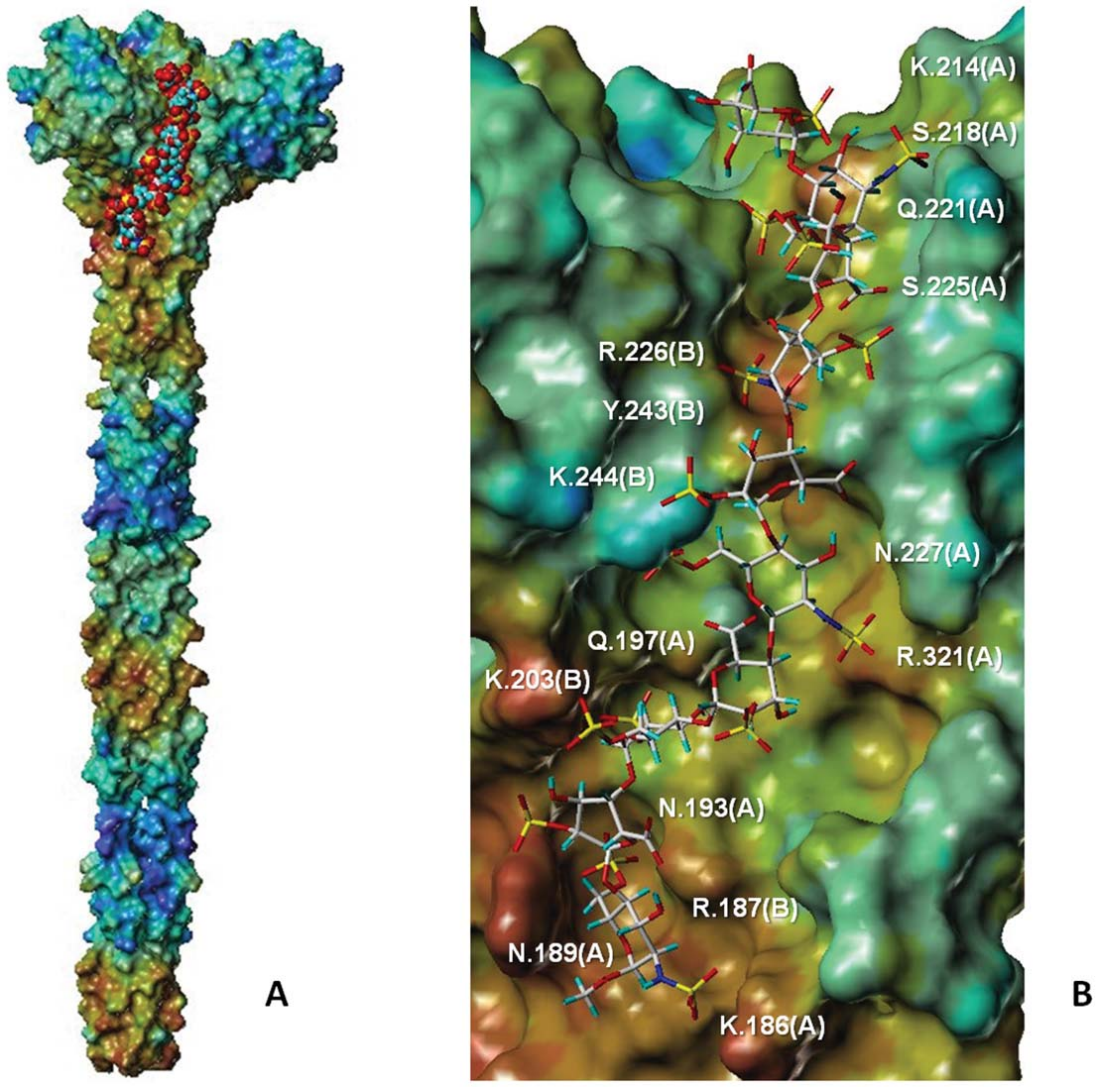
Heparin decasaccharide in complex with Langerin. Predicted binding mode of the heparin decasaccharide (displayed in space fill -A- and capped stick -B-) in complex with Langerin. Langerin is represented with its Connolly surface color-coded according to the electrostatic potential (from blue for negative to red for positive electrostatic areas). Amino acids mediating the main interactions with the decasaccharide are labeled in white.

Docking solutions from the most populated clusters, characterized by reasonable torsion angles and negative (favorable) Autodock scores, were then selected, merged together and minimized. Finally, a decameric Heparin chain was obtained (Figure 5) [25]. Geometrical properties (ring shapes and glycosidic torsions) referred to the heparin bound state are reported in Table 2.

**Table 2:** Details about the geometries of the heparin decasaccharide in complex with Langerin.

		φ = O5_i_ – C1_i_ – O1_i_ – C4_j_	ψ = C1_i_ – O1_i_ – C4_j_ – C5_j_
IdoA*- GlcNAc	Torsion angle 1	−107.3	−163.4
GlcNAc – IdoA°	Torsion angle 2	135.8	−89.3
IdoA°- GlcNAc	Torsion angle 3	−78.4	175.1
GlcNAc - IdoA°	Torsion angle 4	36.5	−169.3
IdoA°- GlcNAc	Torsion angle 5	−65.6	−96.0
GlcNAc – IdoA*	Torsion angle 6	63.3	−76.4
IdoA*- GlcNAc	Torsion angle 7	−56.3	−89.4
GlcNAc - IdoA°	Torsion angle 8	66.2	−168.7
IdoA°- GlcNAc	Torsion angle 9	−75.1	−112.8

The torsion angles were defined as follow: φ = O5_i_ – C1_i_ – O1_i_ – C4_j_ and ψ = C1_i_ – O1_i_ – C4_j_ – C5_j._ The numbering for the torsion angles is reported in Figure1.

(*)L-Idopyranoside monosaccharides in ^1^C_4_. (°)L-Idopyranoside monosaccharides in ^2^S_O_.

**Figure 6:**
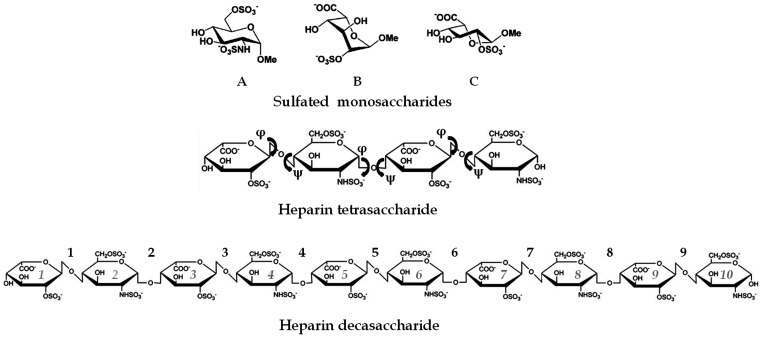
Heparin fragments. 2-N-sulfated, 6-O-sulfated α-D-glucopyranoside (A) and 2-O-sulfated β-L-*idopyranoside* monomers in its ^1^C_4_ (B) and ^2^S_O_ (C) ring shapes were considered for building heparin fragments for docking calculations. Glycosidic linkages are also indicated, defined as φ = O5_i_ – C1_i_ – O1_i_ – C4_j_ and ψ = C1_i_ – O1_i_ – C4_j_ – C5_j._

The bound geometry of Heparin decasaccharide is predicted to adopt a double S-shape conformation, which allows for electrostatic interactions and hydrogen bonds with basic and other polar residues, involving two monomers of Langerin (Figure 5B). The electropositive niches of the protein accommodate well the corresponding charged chemical partners of the decamer. In particular, the presence of two sulfate groups on each GlcNS(6S) residue of Heparin seems to reinforce the binding by playing a *bridging* role between both proteic chains. The GlcNS(6S) residue at the reducing end, for example, is trapped in salt bridges involving the negatively charged oxygen of the sulfate at position 6 and the side chain of Arg187 (Langerin chain B, Figure 5B). The same residue is characterized by a salt bridge formed between the oxygen of the sulfate at position 2 and the Lys186 side chain (Langerin chain A, Figure 5B). Similar situations were found for the GlcNS(6S) residues, labelled 2 and 8 in Figure 6.

A more detailed analysis of the reformed complex (Table 3) brings to light the importance of the O2 sulfation of GlcNS(6S) residues: all of them are implicated in salt bridges or hydrogen bonds with polar aminoacids of Langerin. Indeed, one can assume that O6 is mandatory for stabilizing the complex, beside the key-bridging role of such functional groups, as described previously. The corresponding charged groups of IdoA(2S) residues also favourably influences the complex formation.

**Table 3:** Model of Heparin-Langerin complex: amino acids mediating the main interactions with the decasaccharide.

Heparin Monomers	Atom/Groups (Heparin)	Aminoacid residues (Langerin)
IdoA* (1)	−SO(2)	Lys 214
GlcNAc (2)	−SO(2)	Ser218
	−SO(2)	Gln221
	−SO(6)	Lys214
IdoA° (3)	−COO^-^	Ser225
GlcNAc (4)	−SO(2)	Arg226
	−SO(2)	Tyr243
IdoA° (5)	−O5	Lys244
	−COO^-^	Asn227
GlcNAc (6)	−SO(2)	Arg321
	−SO(2)	Asn227
IdoA* (7)	−COO^-^	Gln197
GlcNAc (8)	−SO(2)	Lys203
	−SO(6)	Gln197
IdoA° (9)	−SO(2)	Arg187
GlcNAc (10)	−SO(2)	Lys186
	−SO(6)	Arg187
	−SO(6)	Asn189

## Discussion

As a C-type lectin receptor of dendritic cells, Langerin is considered as an adhesion or pathogen recognition receptor. However, the real function of Langerin remains elusive, mainly because its natural ligands are still unidentified. The only roles ascribed to Langerin are an implication in HIV binding [26] and an essential involvement in the formation of Birbeck granule (BG) in Langerhans cells [2], [27]. However, the function of such granules keeps being a matter of debate, since Langerin knock-out mice lacking BGs do not display any phenotypic discrepancies (no abnormal response to *Mycobacterium tuberculosis* and *Leishmania major* infection nor to chemical induced skin carcinogenesis) [28].

Up to now, the only positive roles associated with Langerin and BGs in the literature is the ability of the lectin to bind and internalize HIV into BGs, thereby contributing to viral clearance [3]. Finally, it has been shown in LCs that Langerin, along with CD1a, is also involved in the induction of cellular immune responses to *Mycobacterium leprae*, through the presentation of a non peptide antigen and a possible uptake *via* BGs [29]. This reinforced the previous suggestions that BGs could be a non-classical antigen-processing pathway [30].

Here, we have first characterized at the molecular level the interaction between Langerin and HIV envelope glycoprotein gp120. Between the single CRD domain and the whole extracellular domain comprising 3 CRDs, an apparent 100-fold rise of relative binding affinity is observed. This avidity effect, observed here for Langerin oligomeric form, is well known amongst CLRs. More interesting is the *K_d_* of 25µM, measured for gp120 interaction with monomeric CRD. As reported for many other CLRs, CRDs usually exhibit millimolar affinity for monovalent sugars or when recognition occurs only through an oligosaccharide terminal sugar. A *K_d_* of 25 µM suggests that a more extended binding must occur between gp120 glycan and Langerin CRD. Thus, Langerin does not solely bind the terminal mannose of the high mannose present on gp120 but rather a larger oligomannose motif. Finally, the EC_50_ of 282 nM for the Lg ECD/gp120 interaction is in good agreement with the apparent *K_d_* reported for a Langerin/gp140 interaction in another recent study [31].

Apart from oligomannose, Langerin has been shown to have a rather unique specificity, amongst CLRs, towards sulfated sugars [20]. Among putative physiological ligands, keratan sulfate (KS) has been proposed [21]. Indeed, KS is constituted by a repetition of LacNAc motif (Galβ1-4GlcNAcβ1-3)_n_ that can be either sulfated on the C6 of the galactose or on the GlcNAc. As shown by Tateno et al [21], the affinity of Langerin for KS seems to be mainly related to its sulfation content and more particularly regarding galactose C6 sulfation ([6-SO_4_]Galβ1-4GlcNAc). Reported improved binding upon de-sialylation of KS also suggests recognition through the terminal [6-SO_4_]Gal at the Langerin Ca^2+^ binding site. However, KS does not constitute the major GAG potentially encountered by Langerhans cells in epithelium and mucosal tissues [32]. Considering this affinity of Langerin for sulfated glycans together with the Langerhans cell location and migrating properties, we decided to define Langerin binding properties towards a broader range of GAGs. Emphasis was more particularly given to HS (and structurally related heparin), which is abundant in epithelium and mucosa, and directly exposed at dendritic cells surfaces where it participates to the capture of many pathogens as well as immune activation [33], [34].

From then, we went from surprise to surprise. First of all, Langerin is able to bind heparin, HS but also several types of CS. Secondly, this binding can be independent of Ca^2+^ as shown by interaction studies performed in the presence of EDTA. Thirdly, affinities for heparin and heparan sulfate are in the nanomolar range, ranking them as the best ligands ever described for Langerin. Last, but not least, the interaction is strictly dependent upon oligomerization and absolutely not detectable with a single monomeric CRD. From all these points, Langerin/GAG interaction appears to be completely different from how related CLRs traditionally recognize their ligands. The imperious requirement for Langerin trimeric form suggests the existence of a unique binding site constituted by the assembly of at least 2 of 3 protomers. The nanomolar range affinity may suggest an extended binding site. Moreover, contributions of the sulfate groups through intermolecular electrostatic interactions have also been postulated. We have also studied the interaction of Langerin with other GAGs, using competition approaches. Data obtained clearly showed a selectivity of the lectin for HS-like GAGs (HS and heparin), although Langerin also bound to a much more modest level to CS/DS. Interestingly, we also observed binding selectivity amongst the CS/DS samples tested, Langerin exhibiting the highest binding to CS-C. Comparison of these data to GAG disaccharide analysis showed that binding to Langerin could not simply be attributed to a net charge effect and that specific saccharide features were most likely required. Our results suggest that C6 sulfation as well as iduronic acid strengthen the binding. Moreover, the affinity loss observed for heparin upon HSulf-2 treatment highlights the importance of the C6 sulfate present in the [IdoA(2S)-GlcNS(6S)] motif.

We used the recent crystal structure of the langerin trimer [16] to undertake molecular modeling analysis of Langerin interaction with heparin fragments. Combining the trimeric X-ray structure of a truncated ECD with the previously modeled neck region yielded a reasonably robust model to initiate the search for putative favourable heparin binding regions. Two main areas of interaction with heparin have been identified on the whole Langerin surface through MOLCAD electrostatic potential analysis and EADock DSS cavity detection and blind methylsulfate docking. Thanks to this preliminary dual approach, the more precise Autodock docking of heparin fragments was restricted to those specific areas. Outcome results of these molecular simulations yielded three main conclusions: i) neither methylsulfate nor heparin fragment docking pose interact with the calcium ions; ii) Heparin fragment-Langerin interactions are driven by direct polar forces (salt bridges, hydrogen bonds); iii) the molecular recognition of heparin fragments depends upon more than one Langerin CRD : the most populated docking clusters occupy both CRDs characterizing the edge of the α-helix coiled-coil (Figure 4B, red box).

Building on these clear modelling outputs, it was then possible to construct straightforwardly a heparin decamer *in situ*. In the model, the double sulfation of GlcNS(6S) residues appeared essential for the interaction, acting also as a *bridge* between both CRDs. Globally, the proposed model of heparin/Langerin molecular recognition is in full accordance with the biochemical results. However in order to get an accurate estimation of the free energy of binding and to go further towards a physically relevant description of molecular recognition, molecular dynamics studies would be considered as suitable to take into account the charged and flexible aminoacids coating the binding region. Moreover, the construction of the heparin chain was limited to ten monomers. Modelling of a more extended heparin chain could involve other areas of the protein, for instance at the top of the described region, toward the electropositive *cavity* (Figure 4A) involving Lys 299 and Lys 313 residues [21]. Finally, characterization of the Langerin ECD structural organization in solution by SAXS will be conducted and will help to improve also the model of the protein itself.

The multiple approaches of our work give convergent evidence for a novel binding mode of Langerin ligands. Remarkably, the binding is independent from the canonical Ca^2+^-site. Previously, the existence of two distinct binding sites within Langerin has been postulated on the basis of an X-ray structure of Langerin in complex with maltose. A maltose was described onto the Ca^2+^ but also within a specific cleft present in Langerin CRD only [35]. It finally turns out that the electron density initially attributed to maltose in this large cleft without Ca^2+^ was in fact the C-terminus of an affinity tag coming from a neighboring molecule in the crystal lattice [17]. However, although this initial proposal for a second binding site for carbohydrates, independent of Ca^2+^ was not validated, it finds here, in a different area of the protein, a new revival. Its nature is totally new in CLRs since it represents, as far as we know, the first binding site generated at the interface between two protomers of C-type lectin receptors. Langerin is thus able to selectively interact with sulfated carbohydrate through two totally distinct modes: i) a Ca^2+^-dependent binding mode in the CLR canonical site when OH groups are available in C3 and C4 of the saccharide ring (as for galactose 6 sulfate, for instance) and ii) in a Ca^2+^-independent mode for polysulfated glycans of the GAG family where either C3 or C4 OH groups is engaged in the polysaccharide glycosidic linkage.

Prior to this work, Langerin specificity has already been assessed through several glycan array studies [16], [17], [20], [36]. However, Langerin binding properties towards CS/DS/HS has never been evaluated, as GAGs were missing from the glycan arrays used, except for the work by Tateno *et al.* In that case, heparin -as well as HS, DS, CSA and KS- were present onto the micro array, but only KS, through binding of its terminal saccharide to the canonical binding site, was identified. This result is in apparent disagreement with our present data. However, one likely explanation is that Tateno *et al.* microarray screening was conducted with an Lg-CRD-Fc fusion protein that exhibits the canonical binding site, but not the newly identified GAG binding site described here. This latter one requires the trimeric form of the protein dependant on the presence of the neck region of the extracellular domain of Langerin. This critical observation clearly demonstrates the importance of CLR oligomeric organization, which cannot simply be considered as a sum of independent CRDs. Here, Langerin trimerisation of CRDs also creates a new and unrelated site thanks to the neck domain of the protein.

The identification of the Langerin specificity towards GAGs raises the question of the physiological relevance and role of such an interaction. HS is abundantly present in the tissues hosting Langerhans cells. Surface of dendritic cells themselves exposes proteoglycans bearing long GAG chains. Therefore, it is most likely Langerin will be in contact with GAGs during the life cycle of the Langerhans cell. Interestingly, a previous work studying the biochemistry of LC trafficking pointed out that heparin, and more particularly N-sulfated glucosamine moieties of heparin, could inhibit LC trafficking [37]. Indeed, a heparin binding factor was postulated to be involved in LC migration. Future work will have to examine a possible role of Langerin in the modulation of LC trafficking. Another possibility might be a synergistic implication of both heparin and Langerin in pathogen recognition.

This work highlighted the unique properties of Langerin to interact with glycans through both a Ca^2+^ binding site, as for gp120 high mannose, and a new and never reported GAG specific binding site. This raises many new issues about the physiological role of Langerin within the Langerhans cells.

## Materials and Methods

### Expression and Purification of Recombinant Langerin Domains

Soluble Lg CRD was expressed in the periplasmic compartment and purified as previously described, using a one step Strep-Tag II purification [38]. Langerin ECD was expressed in inclusion bodies. Refolding and purification procedures were performed as already described [27] . After refolding, purification of functional Lg-ECD proteins was achieved by affinity chromatography on a mannan-agarose column (Sigma) equilibrated in buffer A (150 mM NaCl; 25 mM Tris pH 7.8) supplemented with 4 mM CaCl_2_ and eluted in buffer A without CaCl_2_ but supplemented with 10 mM EDTA. This step was followed by a Superose 6 size exclusion chromatography equilibrated in buffer A with 4 mM CaCl_2_.

### Surface Plasmon Resonance

#### Interaction between Langerin and gp120

All experiments were performed on a BIAcore 3000 using CM4 chips and the corresponding reagents from BIAcore. Three different sensor chips were used to study the interaction between Langerin and gp120_YU2_: the first one was used to study the interaction between Lg-S-CRD and gp120_YU2_. For this, two flow cells of a CM4 sensor chip were activated by 50 µL of a mixture EDC/NHS. Flow cell one was functionalized with 10 µg/mL BSA, in 5 mM sodium acetate pH 4.5 buffer (639 RU immobilized), blocked with 50 µL of 1 M ethanolamine and used as a control surface. Flow cell two was functionalized with gp120_YU2_ at 10 µg/mL in 5 mM sodium acetate pH 4.5 buffer to reach an immobilization level of 514 RU. Running buffer was buffer A supplemented with 4 mM CaCl_2_ and 0.005% of P20 surfactant. Binding assays were performed at 20 µl/min, by injecting two-fold dilution series of Lg-CRD (12.5 µM to 400 µM) and Lg-ECD (2 nM to 2 µM) for 2.5 and 12.5 minutes, respectively. Regeneration was achieved by injection of 50 mM EDTA (20 and 50 µl for Lg-CRD and Lg-ECD, respectively). The 1∶1 Langmuir model, included within the BIAeval 3.1 software, was used to fit the Lg-CRDs sensorgrams. Analysis of Lg ECD onto a gp120-immobilized surface was performed similarly. BSA negative control and gp120 surfaces (600 RU and 838 RU immobilized, respectively) were prepared as described above. Two-fold dilution series of Lg-ECD (0,48 nM to 2 µM) in running buffer A plus 4 mM CaCl_2_ and 0.005% P20 were injected over both surfaces at 20 µL/min for 12.5 minutes (750 µL). Regeneration was achieved by injection of 50 mM EDTA for 2.5 minutes.

#### Interaction between Langerin and glycosaminoglycans

Binding of Langerin to GAGs was analysed using direct interaction and competition approaches. For this, three flow cells were activated as described above, and functionalized with streptavidin at 100 µg/mL in 5 mM sodium acetate pH 4.5 buffer. Flow cell one was used as negative control surface. Fifteen kDa and 6 kDa heparins (Sigma-Aldrich) were biotinylated as described previously [39], then immobilized on flow cells 2 and 3 (20 and 30 RU, respectively), by injection at 5 µg/mL in 0.3 M NaCl for 5 minutes. Non-specific binding was removed by injection of 20 µL of 2 M NaCl. Binding assays were performed at 10 µL/min in running buffer A plus 4 mM CaCl_2_, 0.005% P20 or running buffer A plus 1 mM EDTA, 0.005% P20. Regeneration was performed by injection of 350 mM MgCl_2_ for 5 minutes. For direct interaction studies, 2-fold dilution series of Lg-CRD (1.6 to 100 µM) were injected over the surfaces at a 10 µL/min flow rate. For direct interaction studies with Lg-ECD, concentration ranges used were from 0.5 nM to 1 µM in Ca^2+^ buffer and from 0.5 nM to 8 µM in EDTA buffer with 2-fold serial dilution factor. For competition assays, two flow cells of a CM4 sensor chips were functionalized with streptavidin as described above, and 20–30 RU of biotinylated 6 kDa heparin were immobilized onto flow cell 2. The protein concentration used in the injected analyte sample was 500 nM together with different GAGs concentrations from 7.8 nM to 2 µM with 2-fold serial dilution factor.

### Preparation of 6-O-desulfated Heparin and Disaccharide Analysis of GAGs

Vectors pcDNA3.1/Myc-His(-) encoding for HSulf-2 were used to transfect FreeStyle 293-F cells (Invitrogen), using the protocol provided by the manufacturer. 72 h post-transfection, selection of stable transfectants was carried out for 3 weeks by addition of G418 (400 µg/ml). Transfected cell culture supernatants were collected, extensively dialysed against 50 mM Tris pH 7.5, and concentrated 100X by ultrafiltration. HSulf-2 activity was assessed as previously described [40], by incubation of HSulf-2 with 10 mM of fluorogenic pseudosubstrate 4-MUS (Sigma) in 50 mM Tris, 20 mM MgCl_2_ pH 7.5 for 3 hours at 37°C, and measurement of unbelliferone fluorescence (exc. 360 nm, em. 460 nm). Specific 6-O-desulfation of 15 kDa heparin (Sigma) was then performed by incubation with 150 µl of HSulf-2 supplemented with MgCl_2_ (2 mM final concentration) for 24 hours at 37°C. Samples were then heated at 100°C for 5 minutes to terminate the reaction.

Disaccharide analysis of heparin was performed as previously described [41], Briefly, samples were exhaustively digested by successive incubation with heparinase I (10 mIU 24 h at 30°C), then heparinases II and III (10 mIU each, 24 h at 37°C), in 100 mM sodium acetate, 0.5 mM calcium acetate, pH 7.1. Complete digestion into disaccharides was confirmed by analysis of the digestion products in size exclusion chromatography, using twinned Superdex Peptide 10/300GL columns equilibrated in PBS, 0.3 M NaCl and run at 0.5 ml/min. Disaccharides were then resolved by strong anion exchange (SAX)-HPLC (Propac PA1, Dionex) equilibrated in H_2_O pH 3.5, over a 0–1 M NaCl gradient. Peaks were detected by measuring absorbance at 232 nm and elution positions compared to those of authentic disaccharide standards (Iduron).

Disaccharide analysis of CS-A, DS and CS-C (Sigma) was performed similarly. Sample digestion was achieved by incubation in 50 mM Tris-HCl, 50 mM NaCl, 2 mM CaCl2, 0.01% BSA, pH 7.5 with 500 mU of chondroitinase ABC for 24 h at 37°C. Identification of disaccharides by SAX-HPLC was carried out using a 0–0.75 M NaCl gradient calibrated with CS/DS disaccharide standards (Iduron).

### Molecular Modelling

#### Modelling of the Langerin ECD target protein

The Langerin ECD structure was built by merging the crystal structure of the Langerin trimer taken from the Brookhaven Protein Data Bank (PDB ID: *3kqg*) with the trimeric coiled-coil neck region derived from our previously published homology model [16], [27], Hydrogen atoms were added and Gasteiger-Huckel partial atomic charges were assigned. Titratable groups were considered in their standard protonation state at neutral pH. Orientation of hydrogen atoms were optimized by using the AMBER force field as implemented in the SYBYL8 package (Tripos Inc., St Louis, MO).

#### Surface mapping and EADock runs: identification of potential heparin interacting areas on the Langerin ECD target protein

Connolly surface was computed by the MOLCAD program [42] around the modelled Langerin ECD structure. Electrostatic potential was mapped and visualized within the SYBYL graphical environment. The EADock DSS docking methodology was used through the SwissDock web service [24], [43] (http://www.swissdock.ch) to predict the most favourable anchoring positions for the negatively charged moieties of heparin at the Langerin surface. A methylsulfate input fragment was considered herein as a probe for blind docking simulations in the *accurate* mode setting. The protein was considered as rigid. Since this simulation aimed mainly at considering the various areas important for intermolecular electrostatic recognition forces, assigned charges were carefully checked. Indeed the MMFF94 force field [44] delocalizes charges on the sulfate group correctly (−0.8167 on each oxygen atoms and +1.6337 on the sulfur) and the CHARMM22 force field [45] treats the calcium ions of Langerin properly by assigning a +2.0 charge per atom. Four runs, each one yielding 250 docking solutions, were performed and docking poses were clustered for evaluation at 2Å-rmsd.

#### Heparin fragments: constructions and parameterization

XYZ coordinates of 2-N-sulfated, 6-O-sulfated α-D-Glc and 2-O-sulfated β-L-IdoA monomers were retrieved from the Monosaccharide databank available at www.cermav.cnrs.fr/cgi-bin/monos/monos.cgi. Heparin fragments were then generated by alternating such monomers to form heparin tetramers, also considering the occurrence of ^2^S_O_ and ^1^C_4_ ring shapes for IdoA (Figure 4). Partial charges were assigned according to the PIM force field [46] whereas geometries were adjusted through conjugate gradient energy minimization within the Tripos force field [47].

#### Heparin fragment and the Langerin ECD target protein: molecular docking

Heparin fragments were considered as ligands for docking performed by the AutoDock 3.0 program [48]. This methodology is widely considered suited for carbohydrate-protein molecular recognition and, in particular, for those involving glycosaminoglycans [49]. Langerin target structure and heparin fragments were herein modeled with explicit hydrogen atoms, protonated as at pH 7.4. Gasteiger-Huckel and PIM partial atomic charges were assigned to the protein and to the ligands, respectively. The three calcium atoms located at the edge of Langerin were considered as separated bodies by assigning the formal charge +2 and Lennard-Jones parameters as described in our previous work [27]. The most favored area where heparin interactions may take place, identified through the surface mapping analysis and EADock DSS runs, was considered as potential binding site, thus enclosed in 0.4 Å-lattice grids (160 Å×100 Å×100 Å).

During Autodock runs the protein was considered as a rigid body whereas all the rotatable bonds of the fragments were flexible, allowing the glycosidic linkages of the heparin units to adopt different yet realistic conformations. For each fragment, 50 docking solutions were retrieved after Lamarckian Genetic Algorithm cycles, each one characterized by a generation of 50 individuals and 1×10^6^ energetic evaluations. Since water was not modeled explicitly, a dielectric constant of 4 r was accounted. Finally, 2.5 Å-rmsd clusters of docking solutions were established and analyzed.

#### Modeling of the heparin decamer in complex with Langerin ECD target protein

An extended heparin chain was obtained by merging representative saccharidic fragment docking solutions from Autodock population clusters. The heparin chain, consisting of 10 monosaccharides, was modeled according to the energetically accessible glycosidic linkage information available in literature [25]. Heparin-Langerin reformed complex was then submitted to staged-energy minimization cycles of 1000 iterations each, by first involving hydrogen atoms and side chain residues of Langerin. Then the entire complex was free to relax. All the minimization cycles were performed within the Tripos force field added with PIM parameters, dedicated to carbohydrates [46]. The permittivity was set as a distance-dependant function and a Powell-type minimizer was used through the calculations.
